# Polarized Cell Division of *Chlamydia trachomatis*

**DOI:** 10.1371/journal.ppat.1005822

**Published:** 2016-08-09

**Authors:** Yasser Abdelrahman, Scot P. Ouellette, Robert J. Belland, John V. Cox

**Affiliations:** 1 Department of Microbiology, Immunology, and Biochemistry, University of Tennessee Health Science Center, Memphis, Tennessee, United States of America; 2 Department of Microbiology and Immunology, Faculty of Pharmacy, Cairo University, Cairo, Egypt; 3 Division of Basic Biomedical Sciences, Sanford School of Medicine, University of South Dakota, Vermillion, South Dakota, United States of America; University of Massachusetts Medical School, UNITED STATES

## Abstract

Bacterial cell division predominantly occurs by a highly conserved process, termed binary fission, that requires the bacterial homologue of tubulin, FtsZ. Other mechanisms of bacterial cell division that are independent of FtsZ are rare. Although the obligate intracellular human pathogen *Chlamydia trachomatis*, the leading bacterial cause of sexually transmitted infections and trachoma, lacks FtsZ, it has been assumed to divide by binary fission. We show here that *Chlamydia* divides by a polarized cell division process similar to the budding process of a subset of the Planctomycetes that also lack FtsZ. Prior to cell division, the major outer-membrane protein of *Chlamydia* is restricted to one pole of the cell, and the nascent daughter cell emerges from this pole by an asymmetric expansion of the membrane. Components of the chlamydial cell division machinery accumulate at the site of polar growth prior to the initiation of asymmetric membrane expansion and inhibitors that disrupt the polarity of *C*. *trachomatis* prevent cell division. The polarized cell division of *C*. *trachomatis* is the result of the unipolar growth and FtsZ-independent fission of this coccoid organism. This mechanism of cell division has not been documented in other human bacterial pathogens suggesting the potential for developing *Chlamydia*-specific therapeutic treatments.

## Author Summary

The obligate intracellular human pathogen *Chlamydia trachomatis* is the leading bacterial cause of sexually transmitted infections. *C*. *trachomatis* does not express FtsZ, which is necessary for the highly conserved process of binary fission that most bacteria utilize to divide. Nonetheless, it has been assumed that this bacterial pathogen divides by binary fission. We show here that *C*. *trachomatis* divides by a polarized cell division process that is similar to the budding process of some other bacteria that lack FtsZ, such as the Planctomycetes. This novel mode of cell division has not been documented in other human bacterial pathogens suggesting the potential for developing *Chlamydia*-specific therapeutic treatments.

## Introduction

The genus *Chlamydia* has been placed in the *Planctomycetes-Verrucomicrobiae-Chlamydiae* (PVC) superphylum based on its 16*S* rRNA sequences [1]. The *Planctomycetes* and *Chlamydiae* also lack FtsZ, which is involved in the organization of the septal plane during binary fission in most bacteria [2]. Another feature of members of the family *Chlamydiaceae* is the unique biphasic developmental cycle they undergo during invasion and intracellular growth in susceptible cells [3]. The metabolically dormant form of the organism, the elementary body (EB), is capable of infection and entry into cells where it undergoes differentiation into a metabolically-active, but non-infectious form termed a reticulate body (RB) that replicates within an intracellular vesicle termed an inclusion. The EB to RB differentiation process is understudied due to the technical limitations associated with studying a single bacterium within a eukaryotic cell and as a result is poorly understood. Here, we determined the morphological changes that *Chlamydia* undergoes during these early stages of differentiation and during the initial rounds of cell division within infected cells. Electron microscopic studies extending back over 40 years [4] have led to the proposal that chlamydial RBs divide by binary fission. However, our analyses challenge this assumption by showing that asymmetric membrane expansion in the FtsZ-lacking *C*. *trachomatis* results in a polarized mode of cell division that is very similar to the budding process that occurs in a subset of the FtsZ-less *Planctomycetes* [5–8].

## Results

### Asymmetric Membrane and Cytoplasmic Organization and Polarized Cell Division of *C*. *trachomatis*

**Fig 1:**
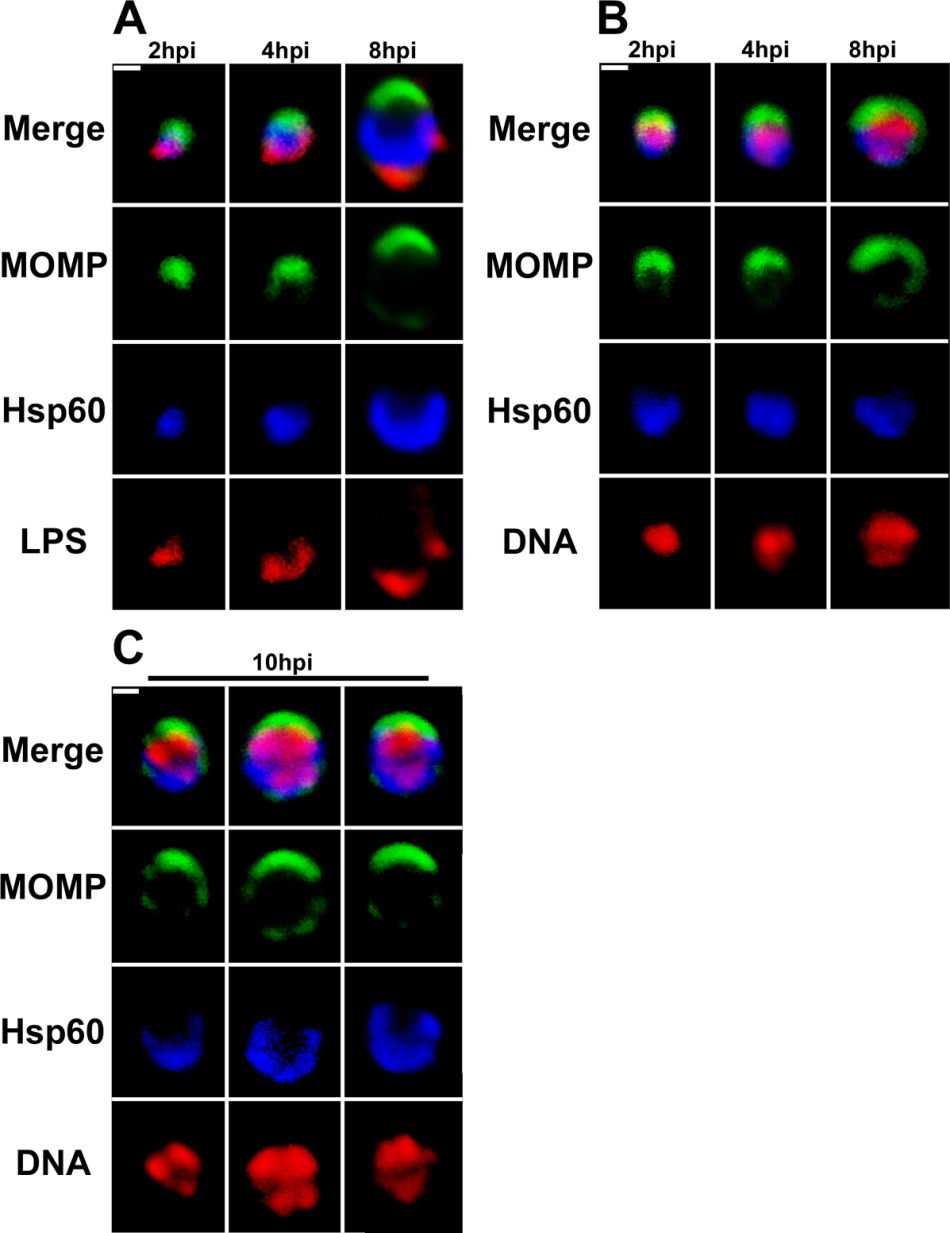
Outer membrane and cytosolic markers are polarized in *C*. *trachomatis* serovar L2. HeLa cells infected with *C*. *trachomatis* were fixed at 2, 4, 8 (A and B), or 10 (C) hours post-infection (hpi). Following fixation, the cells were permeabilized and LPS localization was determined by incubating cells with anti-LPS mouse monoclonal antibodies followed by donkey anti-mouse IgG conjugated to Alexa Fluor 568 (A; red), Hsp60 localization was determined using anti-Hsp60 rabbit polyclonal antibodies followed by donkey anti-rabbit IgG conjugated to Alexa Fluor 633 (A, B, and C; blue), and MOMP localization was determined using anti-MOMP goat polyclonal antibodies followed by donkey anti-goat IgG conjugated to Alexa Fluor 488 (A, B, and C; green). Following the antibody incubations, the cells were imaged by confocal microscopy. (B and C) DNA was visualized by staining with Hoechst 33342 (red). White bars are 0.5μ.

Following invasion of the host HeLa cell, internalized *C*. *trachomatis* undergoes a gradual increase in size between 2 and 8 hours post-infection (Fig 1A and S1 Table). At these early time points, confocal microscopy revealed that the major outer-membrane protein (MOMP) and lipopolysaccharide (LPS) were present on opposite poles of the cell (Fig 1A). Other investigators have shown a similar polarized distribution of the chlamydial type III secretion machinery in pre-entry EBs [9,10] and in differentiating RBs [11]. While MOMP was always present as a patch on one side of the cell at 8 hours post-infection, LPS was often present in distinct clusters at the opposite pole of the cell (Fig 1A). In addition to the polarity exhibited by these outer membrane markers at 8 hours post-infection, the cytosolic chaperone heat-shock protein 60 (Hsp60) accumulated at the same pole of the cell as LPS (Fig 1A). The bacterial chromosome, which was primarily present in a region of the cytosol devoid of Hsp60, also began to exhibit a sub-domain structure at this stage of differentiation (Fig 1B). By 10 hours post-infection, MOMP and Hsp60 double-positive *Chlamydia* had increased to ~1.3μ in diameter (S1 Table) and ~80% of the cells contained 2–4 discrete regions of DNA and a subset of these regions of DNA density were juxtaposed to the MOMP-positive pole of the cell (Fig 1C). Whether these DNA sub-domains reflect intermediates in DNA replication or de-condensation of the chromosome associated with the onset of gene expression in RBs is unclear at this time.

**Fig 2:**
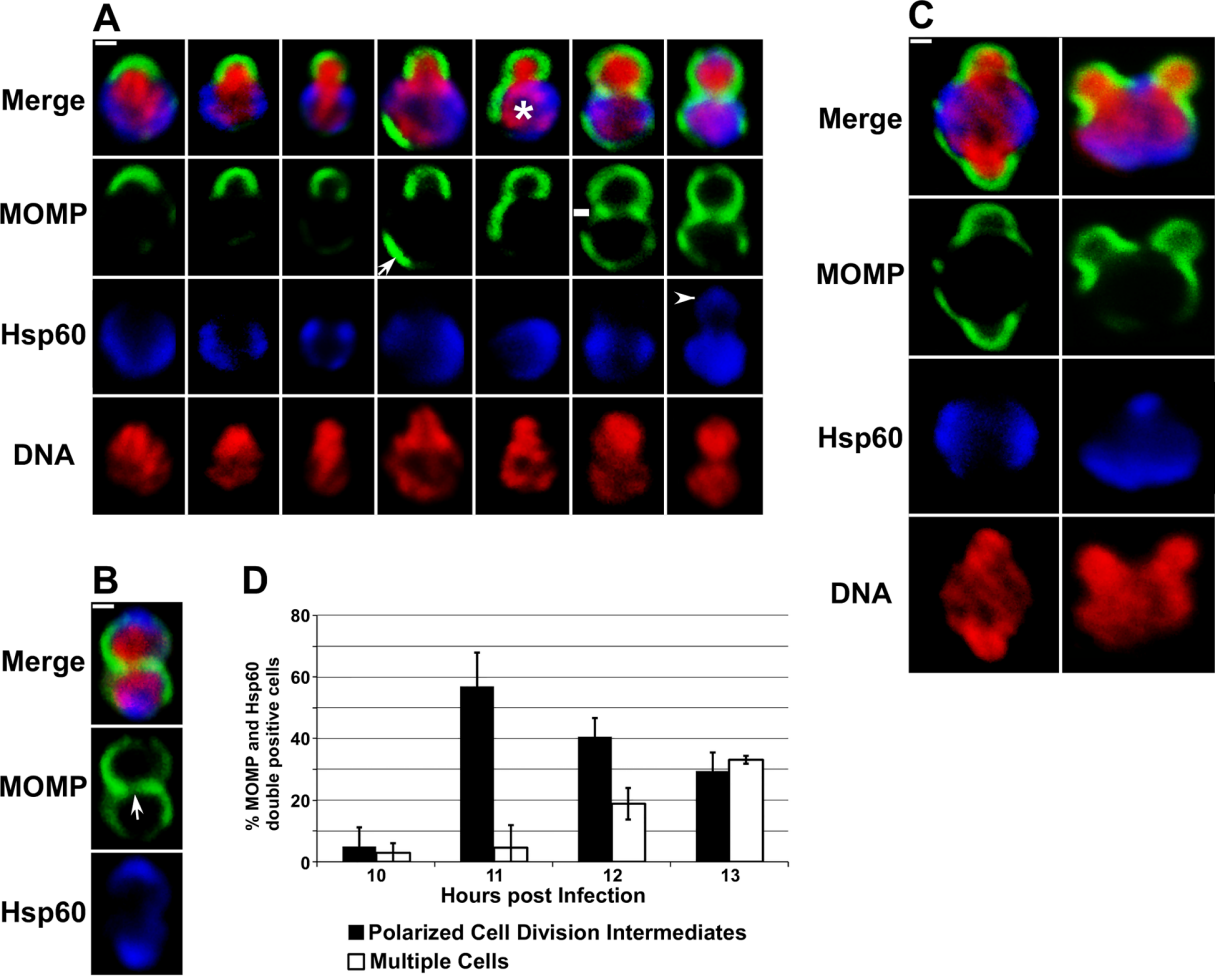
*C*. *trachomatis* divides by a polarized cell division process. HeLa cells infected with *C*. *trachomatis* were fixed at 11 (A and C) or 13 (B) hours post-infection. Following permeabilization, the cells were incubated with rabbit polyclonal antibodies against Hsp60 (blue) and goat polyclonal antibodies against MOMP (green) followed by donkey anti-rabbit IgG conjugated to Alexa Fluor 568 and donkey anti-goat IgG conjugated to Alexa Fluor 488 secondary antibodies. The cells were then washed and stained with Hoechst 33342 prior to confocal analysis. Arrow in A points to a region of polarized MOMP in a mother cell. Dash in A denotes the presence of a MOMP-poor septum separating the mother and daughter cell. Arrowhead in A points to Hsp60 in the daughter cell. (B) Localization profile of MOMP, Hsp60, and DNA in a cell that has completed its first division. Arrow in B points to a MOMP-poor region in the center of the septum between the mother and daughter cell. (C) Examples of *C*. *trachomatis* at 11 hours post-infection that were simultaneously undergoing polar growth from two sites in the cell. (D) Infected HeLa cells were fixed at 10, 11, 12, or 13 hours post-infection and the percentage of *C*. *trachomatis* that were undergoing polarized cell division (black bars) or had completed the first division (white bars) were quantified. All of the MOMP and Hsp60 double-positive cells in randomly selected fields were included in the analysis. White bars are 0.5μ.

The polarity of MOMP observed in *Chlamydia* at early times post-entry was maintained as RBs underwent their first division at ~11 hours post-infection. Unexpectedly, this division process was characterized by an asymmetric expansion of the chlamydial membrane from the MOMP-positive pole of the cell in a process that was very reminiscent of budding in some of the *Planctomycetes* [5–8]. Images in Fig 2A detail the intermediates in membrane reorganization that occur during this division process. The presence of these various cell division intermediates at 11 hours post-infection reflects the asynchronous nature of *Chlamydia* internalization and development. At the earliest stages, the MOMP-positive pole of the cell bends outward and as the daughter cell enlarges the bacterial chromosome appears to be tightly associated with its expanding membrane (Fig 2A). While the bacterial chromosome almost entirely filled the cytosol of the daughter cell, chlamydial Hsp60 was restricted to the cell lacking MOMP, which we refer to as the mother cell (Fig 2A). As the division process continues a new region of MOMP staining (arrow in Fig 2A) appears in the membrane of the mother cell. A polar relationship between MOMP and Hsp60 is maintained within the mother cell as Hsp60 and this new region of MOMP staining reside on opposite sides of the cell (Fig 2A). The movies in S1 and S2 Videos are animations of 3 dimensional projections of cells at different stages of cell division. These movies more clearly illustrate the spatial organization of MOMP, Hsp60, and the chromosome at a relatively early (S1 Video) and at an intermediate stage (S2 Video) of this cell division process. At later stages of cell division when the daughter cell is almost equivalent in size to the mother cell, MOMP becomes less polarized in the mother cell and a MOMP-positive septum appears to separate the dividing cells. Others have reported the presence of MOMP in the septum [12,13], and the daughter cell is still devoid of Hsp60 when a septum containing low levels of MOMP is first detected (marked by a dash in Fig 2A). During later stages of division when additional MOMP accumulates in the septum, Hsp60 begins to accumulate in the daughter cell (arrowhead in Fig 2A). This late stage intermediate in the first division also contained a region in the center of the septum that contained lower levels of MOMP. The MOMP-poor region of the septum is maintained in cells that have completed the first division (arrow in Fig 2B), which exhibit a profile that would be expected if the cells divided by binary fission. At this stage, the mother and daughter cells are the same size and have a similar content and distribution of MOMP, Hsp60, and DNA (Fig 2B).

Approximately 5% of the cells undergoing their first division exhibited polar growth from more than one location in the cell (Fig 2C). The multiple sites of polarized growth that occurred in these cells still emerged from MOMP-rich membrane domains that excluded Hsp60. Our data suggest that the mechanisms that specify the temporal and spatial regulation of sites of polar growth in *C*. *trachomatis* are not tightly controlled. Aspects of the polarized division process of *Chlamydia* are similar to the budding process of a subset of the *Planctomycetes* [5–8].

*C*. *trachomatis* that undergo an increase in size (S1 Table) following internalization into the infected host stain positive for MOMP and Hsp60. In addition, we have not observed cell division in any cells that are not positive for both of these markers. Quantification of all of the MOMP and Hsp60 double-positive *Chlamydia* present in infected cells between 10 and 13 hours post-infection (Fig 2D) indicated that single cells undergoing the polarized cell division process illustrated in Fig 2A are observed prior to the appearance of multiple cells that exhibit the staining profile shown in Fig 2B. As the percentage of polarized cell division intermediates declines at 12 and 13 hours post-infection there is a corresponding increase in multiple cells (Fig 2D). These data indicate that the initial cell division of the majority of *Chlamydiae* within infected cells occurs by a polarized division process. However, we cannot rule out the possibility that a small subset of the initial cell divisions within infected cells occur by binary fission.

**Fig 3:**
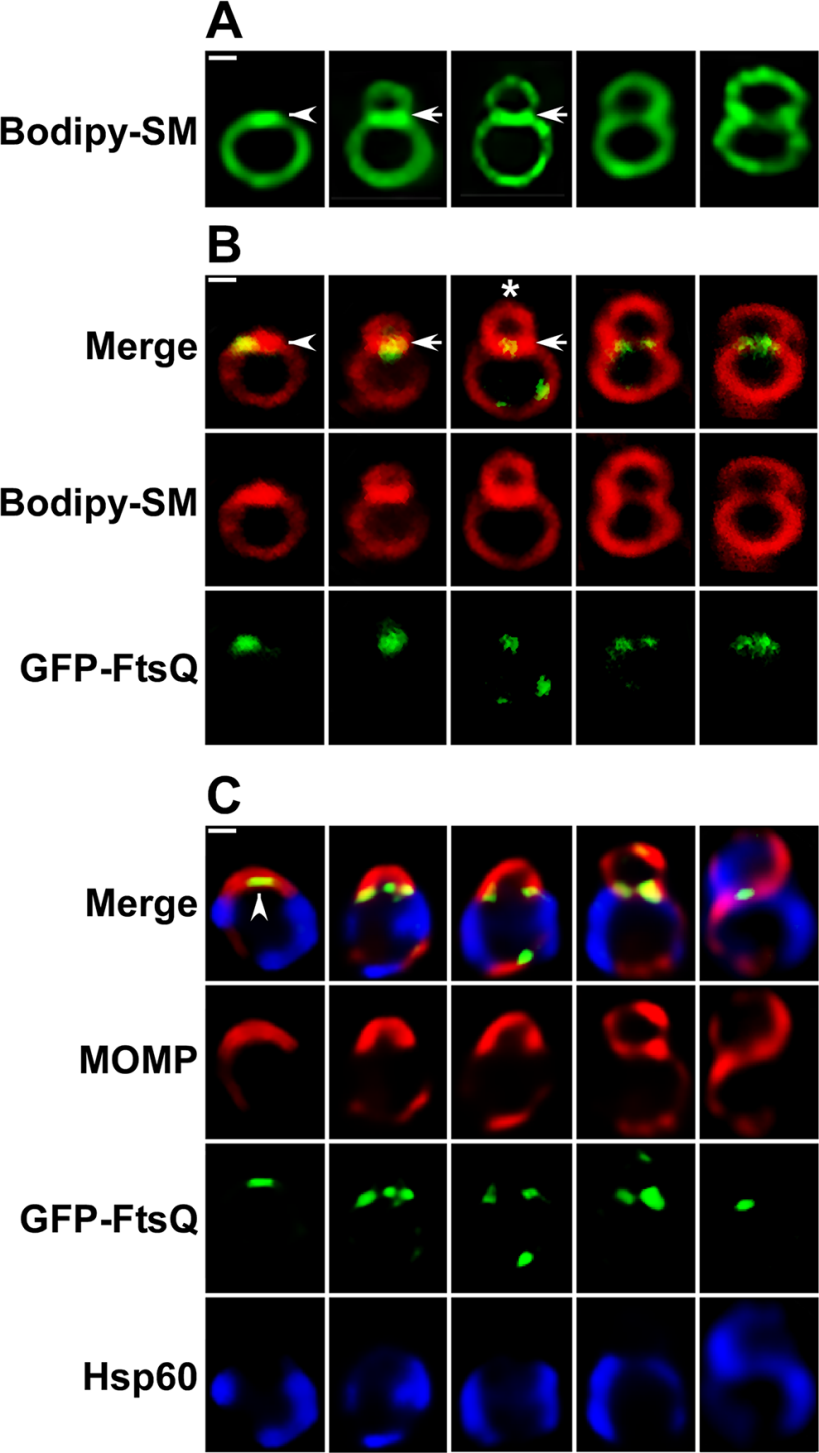
Analysis of the polarized cell division process of *C*. *trachomatis* serovar L2 in live and fixed cells. (A) HeLa cells infected with *C*. *trachomatis* were incubated in the presence of green fluorescent BODIPY FL C5 ceramide as described in the Materials and Methods and polarized cell division intermediates were imaged in live cells at 11 hours post-infection using a Zeiss AxioImager.M2 microscope. (B) HeLa cells were infected with *C*. *trachomatis* serovar L2 that contained an anhydrotetracycline (aTc)-inducible plasmid expressing GFP-FtsQ [17]. aTc was added to infected cultures at 8 hours post-infection then the cells were labeled with red fluorescent BODIPY TR C5 ceramide. Polarized cell division intermediates were imaged in live cells at 11 hours post-infection using a Zeiss LSM710 confocal microscope. Arrowheads in A and B indicate regions of intense BODIPY-sphingomyelin fluorescence at one pole of a round cell prior to division. The polar patch of sphingomyelin fluorescence in B also contained GFP-FtsQ. Arrows in A and B indicate a sphingomyelin-rich membrane separating a nascent daughter cell from a mother cell. (C) HeLa cells were infected with *C*. *trachomatis* serovar L2 that contained aTc-inducible version of GFP-FtsQ. The fusion was induced by the addition of aTc to cultures at 8 hours post-infection and the cells were fixed at 11 hours post-infection. Following permeabilization, the cells were incubated with rabbit polyclonal antibodies against Hsp60 (blue), goat polyclonal antibodies against MOMP (red), and mouse monoclonal antibodies against GFP (green) followed by donkey anti-rabbit IgG conjugated to Alexa Fluor 633, donkey anti-goat IgG conjugated to Alexa Fluor 594, and donkey anti-mouse IgG conjugated to Alexa Fluor 488 secondary antibodies. The cells were then washed prior to imaging on a Zeiss AxioImager.M2 microscope. Note the size and morphology of (B) live and (C) fixed cells are very similar. Arrowhead in C indicates a region of GFP-FtsQ fluorescence in the MOMP-positive pole of the cell prior to cell division.

It was possible that aspects of chlamydial morphology illustrated in Fig 2A may be a consequence of the fixation and permeabilization schemes used in the assays. Therefore, infected HeLa cells were incubated with the fluorescent lipid, BODIPY-ceramide, to determine if *Chlamydia* undergoing this polarized cell division process could be visualized in live cells at 11 hours post-infection. This green fluorescent ceramide analogue is converted to sphingomyelin and delivered to the inclusion where it is incorporated into chlamydial cell membranes [14–16]. The live cell images in Fig 3A demonstrate that polarized cell division intermediates could be visualized in live *Chlamydia* labeled with green BODIPY-sphingomyelin. The size and morphology of the cells labeled with this sphingomyelin analogue (Fig 3A) were virtually identical to the fixed cells in Fig 2A. 3-D projections of all of the Z-slices through asymmetric division intermediates labeled with green BODIPY-sphingomyelin (S1A Fig) further revealed that the images shown in Fig 3A accurately reflect the morphology of dividing cells and are not simply a consequence of the plane of section of the slices through the cells.

An intermediate routinely observed in cells labeled with BODIPY-sphingomyelin contained an intense patch of fluorescence at one pole of a round cell (marked with arrowhead in Fig 3A). This same polar patch of sphingomyelin fluorescence was observed in live cell experiments in which *Chlamydia* were labeled with a red fluorescent version of BODIPY-ceramide (Fig 3B). *C*. *trachomatis* that were labeled with this red ceramide analogue also expressed a tetracycline-inducible version of the cell division protein, GFP-FtsQ [17], and this GFP fusion accumulated within the intense polar patch of red BODIPY-sphingomyelin fluorescence (marked with arrowhead in Fig 3B). Whether the intense polar patch of BODIPY-sphingomyelin fluorescence reflects steps in membrane synthesis necessary for the initiation of polarized cell division is not known at this time. The next intermediates observed in cells labeled with both green and red BODIPY-ceramide exhibited an asymmetric expansion of the cell membrane producing a nascent daughter cell that was separated from the mother cell by a sphingomyelin-rich membrane (arrows in Fig 3A and 3B). GFP-FtsQ was present in this sphingomyelin-rich septum at this stage of the asymmetric division process (Fig 3B). Although it was not possible to image a single *Chlamydia* undergoing the transition between the first two stages illustrated in Fig 3A and 3B, these data suggest that the accumulation of components of the cell division machinery at one pole of the cell precedes the asymmetric expansion of the membrane necessary to produce the daughter cell. As the division proceeds, the daughter cell increases in size and the intensity of the sphingomyelin fluorescence in the GFP-FtsQ positive septum declines (Fig 3A and 3B) suggesting that changes in the lipid content of the septum occur during this polarized division process. Importantly, we showed that labeling cells with the fluorescent ceramide analogues and the expression of GFP-FtsQ did not have a deleterious effect on the growth of *Chlamydia* and the recovery of infectious organisms from infected HeLa cells (S1B Fig).

To further verify that our fixation scheme does not affect the localization of cellular components, infected cells were fixed at 11 hours post-infection and the localization of GFP-FtsQ was compared to MOMP and Hsp60. This analysis again revealed that the morphology and size of fixed (Fig 3C) and live (Fig 3B) cells are very similar. Furthermore, these studies indicated that GFP-FtsQ accumulated in the MOMP-positive pole of the cell prior to division (marked by arrowhead in Fig 3C) and identical to what was observed in live cells, this GFP fusion was present in the septum throughout the polarized division process. Finally, it is clear that MOMP is recruited to the septum between dividing cells relatively late in the division process.

**Fig 4:**
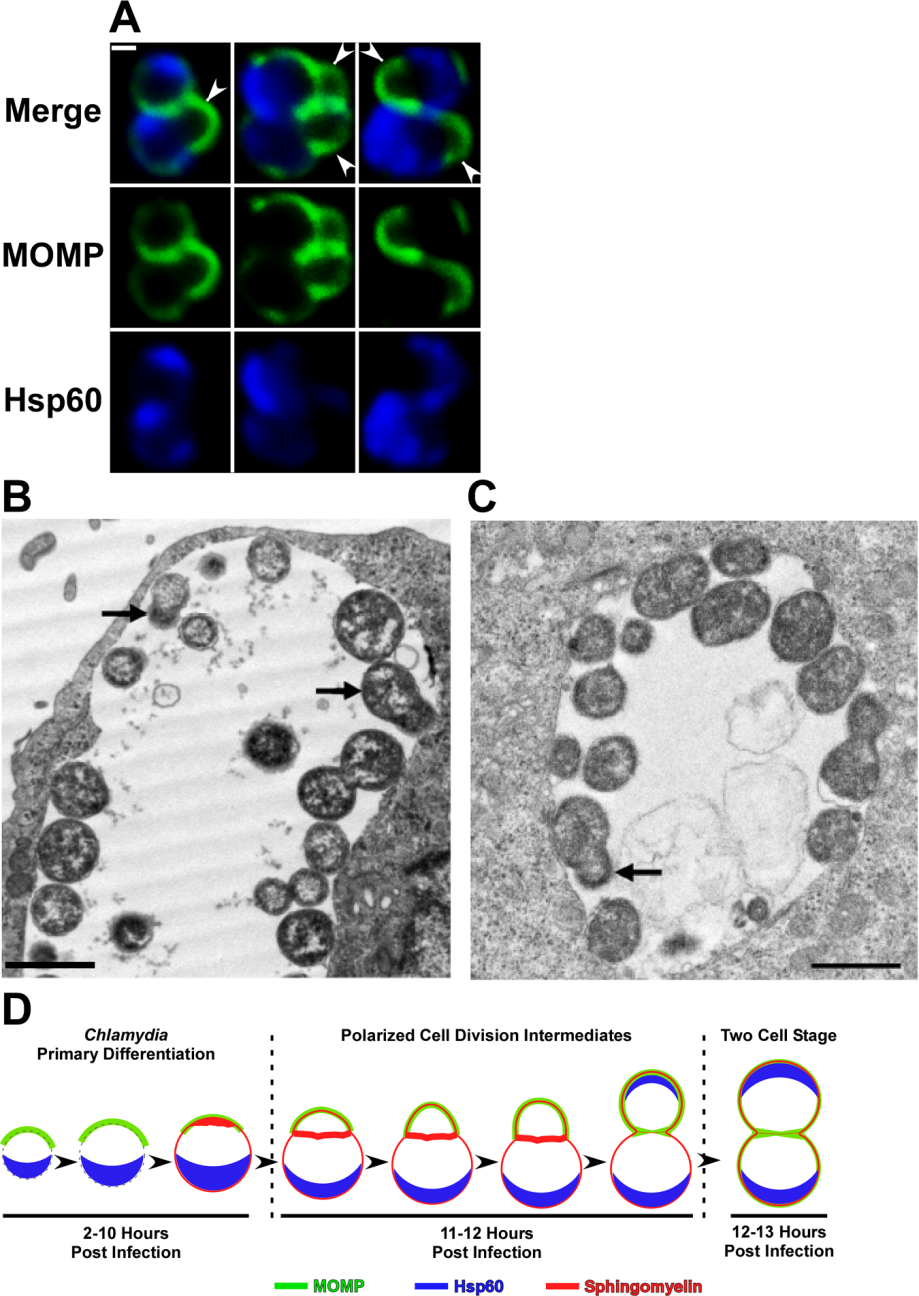
Confocal and EM analysis of the polarized cell division process of *C*. *trachomatis* serovar L2. (A) HeLa cells were infected with *C*. *trachomatis* and fixed at 13 hours post-infection. Following permeabilization, the cells were incubated with rabbit polyclonal antibodies against Hsp60 (blue) and goat polyclonal antibodies against MOMP (green) followed by goat anti-rabbit IgG conjugated to Alexa Fluor 633 and donkey anti-goat IgG conjugated to Alexa Fluor 488 secondary antibodies. The cells were then washed prior to confocal analysis. Arrowheads in A point to sites of polarized growth in cells undergoing the second round of division. (B and C) Alternatively, cells were fixed at 17 hours post-infection and processed for transmission electron microscopy. Black arrows in B and C point to cells undergoing polarized cell division. (D) Model depicting the morphological changes that occur during the primary differentiation and the initial cell division of *C*. *trachomatis* are illustrated. The localization of MOMP (green), Hsp60 (blue), and sphingomyelin (red) is shown. The distribution of sphingomyelin has not been determined in the two earliest intermediates in this model (left hand side of D). The chlamydial membrane is depicted by a dashed line in these intermediates. White bar in A is 0.5μ. The black bars in B and C are 1μ.

The second round of cell division of *Chlamydia* within an infected cell also occurs by a polarized division process. The images in Fig 4A illustrate that the daughter cells of the first division re-establish MOMP and Hsp60 polarity and initiate a second round of division prior to their detachment from each other by undergoing expansion from their MOMP-rich poles. This second division was almost perpendicular to the first division plane. Quantification of cells undergoing their second division revealed three distinct cell division phenotypes that occurred in approximately equal frequency. One-third of the population exhibited an asymmetric expansion of the membrane in only one of the daughter cells, one-third of the daughter cells initiated polarized growth at the same time and in the same direction, and the remaining one-third initiated polarized growth at the same time and in opposite directions (sites of asymmetric growth marked by arrowheads in Fig 4A). Sites of polar growth in this second round of division, like the first division, emanated from the MOMP-rich pole of the cell, but specifying the two different locations where growth initiates in the second division appears to be a random process.

Additional analyses were carried out to determine if we could document this polarized division process using conventional transmission electron microscopic techniques. The EM images in Fig 4B and 4C, which show portions of inclusions in infected cells at 17 hours post-infection, illustrate cells that appear to be undergoing polarized cell division. The cells marked with arrows in Fig 4B contain electron dense material in their putative daughter cells consistent with our observation that the bacterial chromosome almost entirely fills the cytosol of the nascent daughter cell (Fig 2A). An additional cell that appears to be undergoing polarized division is shown in Fig 4C. The membrane of the putative daughter cell (marked by arrow in Fig 4C) exhibited different staining properties than the remainder of the cell. Although the basis for this differential staining is unclear, it may reflect the polarity of cellular proteins we observe in dividing RBs by confocal microscopy. While we cannot rule out that the polarized cell division intermediates shown in Fig 4B and 4C are simply a consequence of the plane of section through dividing *C*. *trachomatis*, EM studies of other investigators have also documented *C*. *trachomatis* undergoing what appears to be polarized cell division [18,19]. S1C Fig contains a magnified version of the cell marked by an arrow in Fig 4C adjacent to magnified versions of the cell division intermediates marked by asterisks in Figs 2A and 3B. The very similar morphologies of these cells lead us to propose that the polarized cell division intermediates detected in our EM studies and those of others arose by the process illustrated in the images in Figs 2 and 3. These early steps in chlamydial differentiation and cell division are illustrated in the model in Fig 4D.

**Fig 5:**
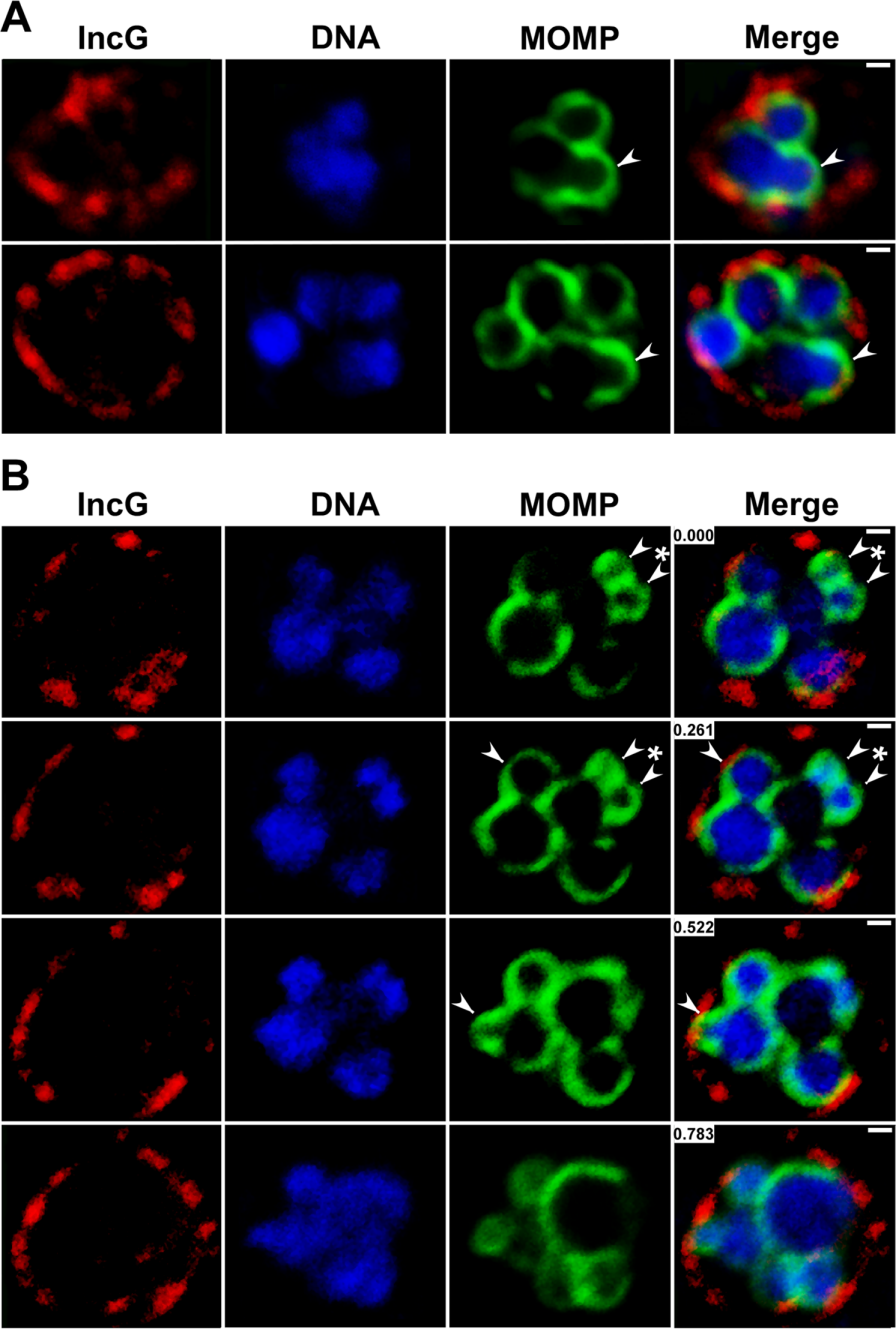
Characterization of the polarized division process of *Chlamydia* in more mature inclusions. HeLa cells were infected with C. trachomatis and fixed at 14 hours post-infection. The cells were then permeabilized and incubated with anti-MOMP goat polyclonal antibodies (green) and anti-IncG rabbit polyclonal antibodies (red) followed by donkey anti-goat IgG conjugated to Alexa Fluor 488 and donkey anti-rabbit IgG conjugated to Alexa Fluor 568 secondary antibodies (A and B). The cells were then washed and stained with Hoechst 33342 (blue) prior to confocal analysis. The images in B are consecutive confocal slices from a z-stack. Arrowheads in A and B point to sites of asymmetric membrane expansion. Asterisk in B indicates a cell simultaneously undergoing polar growth from two sites. The white bars are 0.5μ. The numbers in the merged images in B correspond to the position in the Z-stack in microns.

Polarized cell division intermediates in inclusions containing 6–8 cells were also readily detected by confocal analyses. These dividing cells (indicated by arrowheads in Fig 5A and 5B), like those observed for the first two divisions, were characterized by an asymmetric expansion of the MOMP-rich pole of the cell and a close association of the bacterial chromosome with the expanding membrane of the nascent daughter cell. The consecutive confocal slices from the z-stack shown in Fig 5B illustrate multiple dividing cells within a single inclusion. One of the dividing cells within this inclusion is undergoing simultaneous growth from two sites (marked by asterisk in Fig 5B). A feature of the first few cell divisions that has not been clearly documented in EM studies is the initiation of a second round of cell division in daughter cells that have not yet separated. While a variety of explanations could account for this observation, analyses in which 2-cell stages were stained with antibodies directed against the inclusion membrane protein, IncG, revealed that the inclusion membrane is wrapped very tightly around the cells (S1D Fig). A similar result was observed in the multi-cell inclusions shown in Fig 5A and 5B. These results suggest that the failure of the daughter cells to detach prior to initiating another round of cell division may simply be the result of a rapid rate of cell division coupled with limited space within the inclusion lumen at this early stage of the developmental cycle.

**Fig 6:**
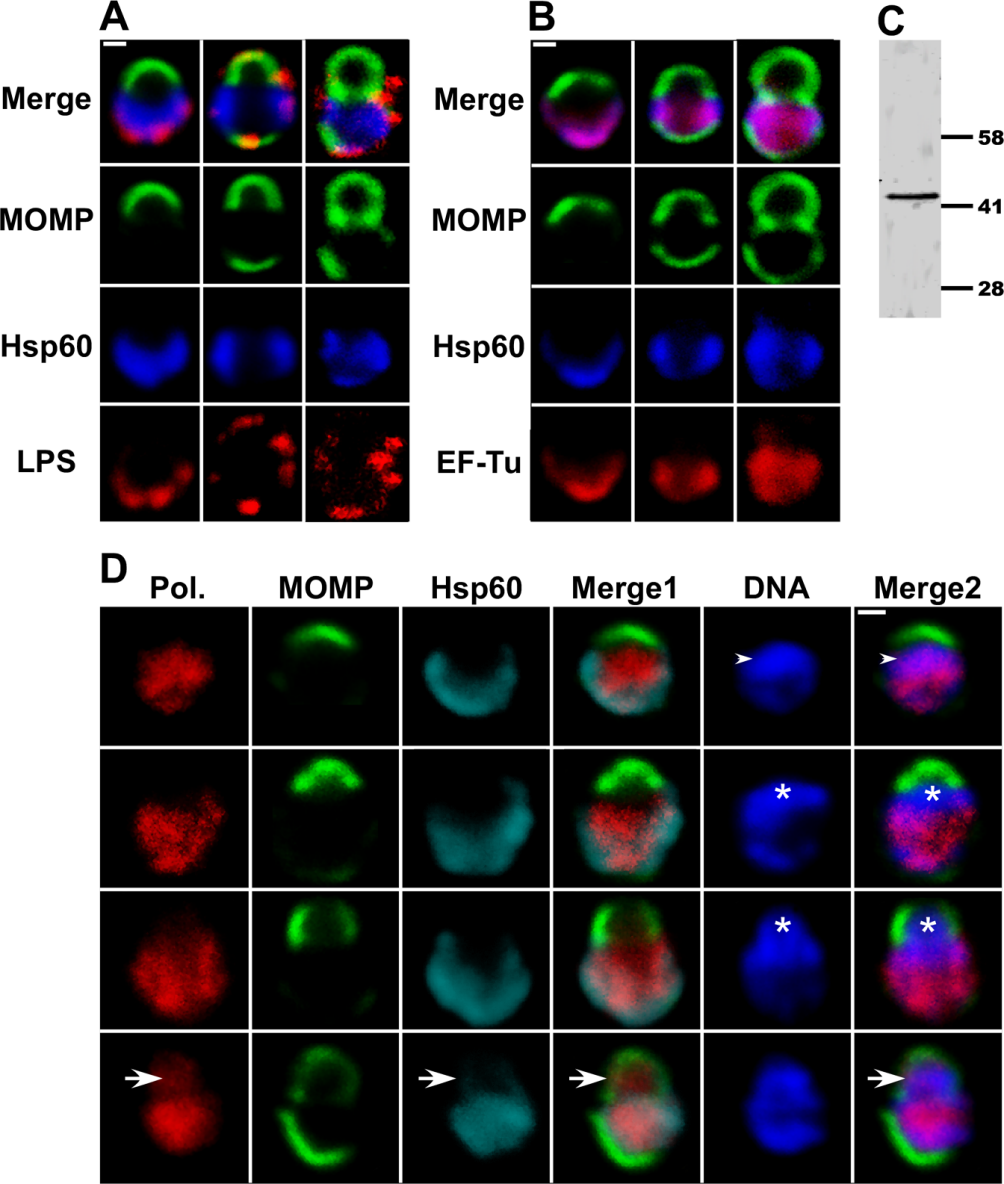
Localization of cytosolic and membrane markers during the polarized cell division process of *C*. *trachomatis*. (A, B, and D) HeLa cells were infected with C. trachomatis serovar L2 and fixed at 11 hours post-infection. The cells were then permeabilized and LPS localization was determined by incubating cells with anti-LPS mouse monoclonal antibodies (A; red), RNA polymerase β subunit localization was determined using mouse monoclonal antibodies (Pol. in D; red), EF-Tu localization was determined using anti-EF-Tu mouse polyclonal antibodies (B; red). The distribution of the mouse primary antibodies was visualized by incubating cells with donkey anti-mouse IgG conjugated to Alexa Fluor 568. Hsp60 localization was visualized using anti-Hsp60 rabbit polyclonal antibodies followed by donkey anti-rabbit IgG conjugated to Alexa Fluor 633 (A and B—blue; D—cyan) and MOMP localization was visualized using anti-MOMP goat polyclonal antibodies followed by donkey anti-goat IgG conjugated to Alexa Fluor 488 (A, B, and D; green). In some instances, the cells were stained with Hoechst (D; blue) prior to confocal analysis. Each panel contains various intermediates in the polarized cell division process. Arrowheads in D denote regions in a nascent daughter cell where the DNA and the RNA polymerase β subunit co-localize. Asterisks in D mark nascent daughter cells that contain DNA but lack the RNA polymerase β subunit and Hsp60. Arrows in D point to region where the RNA polymerase β subunit and the bacterial chromosome co-localize in a nascent daughter cell that does not yet contain Hsp60. In Panel D, Merge1: merge of MOMP/ RNA polymerase β subunit/Hsp60; Merge2: merge of MOMP/ RNA polymerase β subunit/DNA. White bars in A, B, and D are 0.5μ. (C) A lysate prepared from HeLa cells infected with *C*. *trachomatis* at 24 hours post-infection was subjected to immunoblotting analysis with the EF-Tu antibodies. A single 43kDa species that matches the predicted molecular mass of EF-Tu was observed.

Confocal studies also determined whether other membrane and cytosolic elements exhibited a polarized distribution in *C*. *trachomatis* as it undergoes cell division. LPS localization was somewhat heterogeneous in cells undergoing division. In a subset of cells, LPS was restricted to the opposite pole of the cell as the MOMP-rich region undergoing membrane expansion, while in other cells LPS was present in both the mother cell membrane and in a portion of the membrane of the nascent daughter cell (Fig 6A). The cytosolic proteins that were analyzed exhibited distinct localization profiles during the initial division. The translation elongation factor, EF-Tu, very closely mimicked the localization profile of Hsp60 throughout the division process (Fig 6B). In contrast, the β subunit of RNA polymerase exhibited a diffuse pattern of localization in the cytosol of the mother cell similar to the distribution of DNA in the early stages of polarized cell division, and although it did not initially move into the daughter cell with the chromosome, it appeared in the daughter cell prior to the formation of the MOMP-rich septum (Fig 6D). While the mechanism(s) that controls the appearance of bacterial proteins in the daughter cell are undefined, it is clear that different subsets of proteins can be detected in the daughter cell at distinct stages of the cell division process.

### Inhibitors of Polarized Cell Division

**Fig 7:**
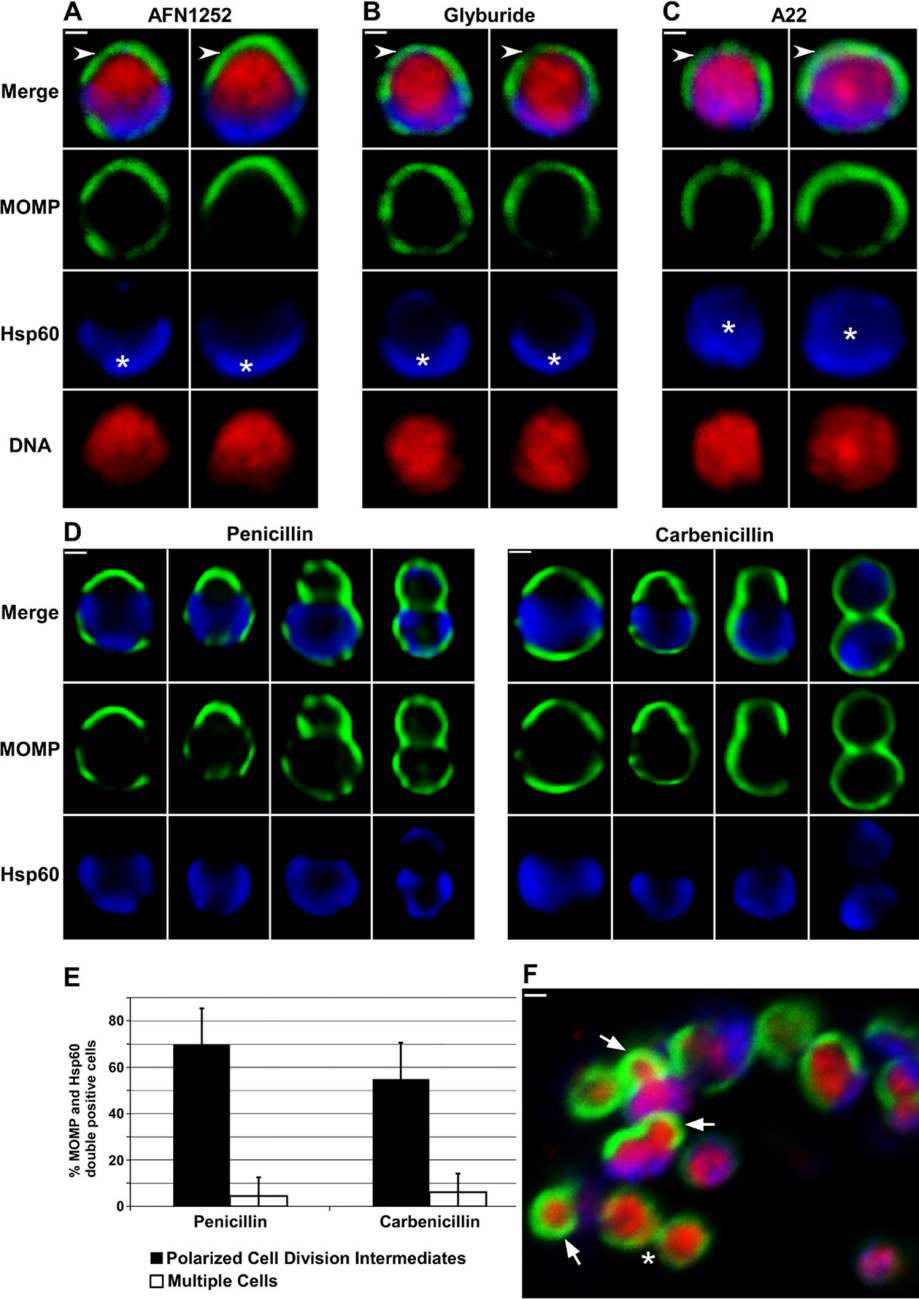
Inhibitors of membrane biosynthesis, peptidoglycan biosynthesis, and MreB prevent the polarized cell division of *Chlamydia*. HeLa cells were infected with *C*. *trachomatis* serovar L2. At 11 hours post-infection, AFN1252 (A), glyburide (B), or A22 (C) was added to the cells and the cells were subsequently fixed at 16 hours post-infection. (D) Penicillin or carbenicillin was added to infected HeLa cells at 10 hours post-infection and the cells were fixed at 12 hours post-infection, or (F) carbenicillin was added to infected HeLa cells at 18 hours post-infection and the cells were fixed 40 minutes later. In each instance, the cells were permeabilized and incubated with rabbit antibodies against Hsp60 (blue) and goat antibodies against MOMP (green) followed by donkey anti rabbit IgG conjugated to Alexa Fluor 633 and donkey anti-goat IgG conjugated to Alexa Fluor 488. In some instances, the cells were stained with Hoechst 33342 (red) prior to microscopic analysis (A—C and F). Two cells illustrating the effect of AFN1252 (A), glyburide (B) and A22 (C) on the polarized cell division process are shown. The cells in A—C are representative of 100 cells that were analyzed from two independent experiments. Panel D illustrates the various intermediates in polarized cell division observed in penicillin-treated or carbenicillin-treated cells. Asterisks indicate polar (A and B) and diffuse (C) Hsp60. Arrows in F point to polarized cell division intermediates observed following carbenicillin treatment of a more mature inclusion containing multiple cells. The asterisk in F indicates a cell that has almost completed division. Images in A-C and F were acquired by confocal microscopy; images in D were acquired by epifluorescent microscopy. White bars are 0.5μ. (E) Penicillin or carbenicillin were added to infected HeLa cells at 10 hours post-infection and the cells were fixed at 12 hours post-infection. The percentage of *C*. *trachomatis* that were undergoing polarized cell division (black bars) or had completed the first division (white bars) were quantified. All of the MOMP and Hsp60 double-positive cells in randomly selected fields were included in the analysis. The data shown represents the analysis of more than 200 cells from two independent experiments.

Inhibitors of membrane biosynthesis, peptidoglycan biosynthesis, and the actin-like protein, MreB [20], have distinct effects on the cell division process of *Chlamydia*. AFN1252 prevents endogenous fatty acid biosynthesis in *C*. *trachomatis* [21] and glyburide inhibits the chlamydial acquisition of phosphatidylcholine from the infected host cell [22]. To further assess the role of these pathways in chlamydial cell division, the drugs were added to infected cells at 11 hours post-infection when ~60% of *C*. *trachomatis* are undergoing their initial polarized cell division (Fig 2D) and the cells were fixed at 16 hours post-infection. Both drugs inhibited the initial cell division and induced the collapse of polarized cell division intermediates, which were absent in drug-treated cells (Fig 7A and 7B). Although Hsp60 retained its polar distribution in drug-treated cells, MOMP exhibited a more symmetric distribution following treatment with AFN1252 (Fig 7A) and glyburide (Fig 7B) suggesting a critical role for endogenous fatty acid biosynthesis and host phosphatidylcholine acquisition in maintaining outer-membrane asymmetry. Analyses with the MreB inhibitor, A22, revealed that this drug blocked cell division as reported previously [20]. Like AFN1252 and glyburide, A22 treatment induced the collapse of polarized cell division intermediates and resulted in a more symmetric distribution of MOMP (Fig 7C). In addition, Hsp60 was more diffusely distributed throughout the cytosol in A22-treated cells suggesting that MreB is necessary for maintaining outer membrane and cytosolic polarity in dividing *C*. *trachomatis*.

Recent studies showed that peptidoglycan accumulates at the point of contact between dividing *C*. *trachomatis* [12] explaining the sensitivity of *Chlamydia* to β-lactam inhibitors [23]. Experiments in which carbenicillin was added to infected cells at 11 hours post-infection and the cells were fixed at 16 hours post-infection confirmed that the polarized cell division of this organism was sensitive to this drug, and in contrast to the drugs described above, carbenicillin treatment did not induce the collapse of polarized cell division intermediates as various intermediates in cell division could be detected (S2A Fig). However, many of the cells began to enlarge and assume aberrant cell shapes during this 5 hour drug treatment making it impossible to quantify the effect of carbenicillin on polarized cell division. To circumvent this problem, we carried out additional analyses in which carbenicillin or penicillin was added to infected cells at 10 hours post-infection prior to the initiation of asymmetric membrane expansion and the cells were fixed at 12 hours post-infection. Although both drugs blocked cell division, they did not prevent asymmetric membrane expansion and a spectrum of polarized cell division intermediates accumulated in drug-treated cells (Fig 7D). Quantification of this analysis revealed that at the concentrations of drugs employed in our assays penicillin appeared slightly more effective than carbenicillin (2% vs. 6%) in preventing the appearance of multiple cells (Fig 7E). The ~70% polarized cell division intermediates observed in penicillin-treated cultures (Fig 7E) was higher than that observed at any stage analyzed in non-drug treated samples (Fig 2D). These data suggest that β-lactam inhibitors arrest cells during their initial polarized cell division. Interestingly, electron microscopic observations by Ouellette et al. [20] of *Chlamydia* treated with β-lactam inhibitors for short times are consistent with these antibiotics arresting the cell division process. While it is likely that these drugs arrest the cells at a specific step in polarized cell division, this step cannot be precisely determined because longer term treatment with the drugs results in cell enlargement and the induction of aberrant cell shapes.

As the number of organisms increased during the developmental cycle, it was technically more challenging to visualize cells undergoing this polarized cell division process. To circumvent this problem, we added carbenicillin to infected cells at 18 hours post-infection for 40 minutes and then fixed the cells for analysis. This approach arrested dividing cells in this more mature inclusion at various stages of the polarized cell division process (marked with arrows in Fig 7F). These results suggest that the process of polarized cell division occurs throughout the chlamydial developmental cycle.

## Discussion

The polarized cell division of *Chlamydia trachomatis* within an infected cell is characterized by a 3-step process of 1) enlargement of a polarized RB, 2) asymmetric expansion of the membrane from the MOMP-rich pole of the cell resulting in the formation of a nascent daughter cell, and 3) FtsZ-independent separation of the mother and daughter cell. The cellular rearrangements that occur during steps 1 and 2 of this cell division process are illustrated in the model in Fig 4D. The asymmetric expansion of the chlamydial membrane that occurs during division results in a pattern of unipolar growth that is very similar to the process of polar growth that occurs during division in some of the Planctomycetes that also lack FtsZ [5–8]. Polarized cell division that is dependent upon DivIVA [24,25] or CdvABC [26] has been described in members of the Actinobacteria and the Archaea, respectively. However, *C*. *trachomatis* lacks obvious homologues of the genes encoding these proteins [27], and the precise mechanism that drives asymmetric membrane expansion in this coccoid organism remains to be defined. Other investigators have recently documented a similar pattern of polarized cell division in *C*. *trachomatis* [28], which may have evolved as a way to specify the septal plane in this FtsZ-less organism.

The pole of the cell that will serve as the site for asymmetric growth during the initial division of *Chlamydia* is enriched in MOMP and this polarized cell architecture is defined at the earliest time point we examined at ~9 hours prior to the first division (Figs 1 and 4D). Immediately following the first cell division, the asymmetric distribution of MOMP was not apparent; however cytosolic Hsp60 maintained its asymmetric distribution at the side of the cell opposite from where polar growth will occur during the second division. The asymmetric expansion of the membrane for division two coincides with the repolarization of MOMP to the site of growth. Additional studies examined the localization of MOMP, LPS, and Hsp60 in purified EBs. MOMP substantially overlapped but was not identical to the distribution of LPS in EBs prior to their entry into HeLa cells (S2B Fig). However, Hsp60 was primarily restricted to a MOMP-poor region of the cell (marked by arrows in S2B Fig). The relationship between polarized Hsp60 and the polarized type III secretion machinery in EBs [9,10] is unclear at this time.

Although *C*. *trachomatis* divides in an FtsZ-independent fashion, it appears to utilize at least some of the machinery required for the binary fission of other bacteria. We and others have shown that known elements of the bacterial cell division apparatus including FtsQ [17], MreB [20,29], RodZ [29], and peptidoglycan[12], are recruited to the septum during chlamydial cell division. Our real-time imaging studies (Fig 3B and 3C) have further shown that FtsQ accumulates in the MOMP-rich pole of the cell prior to the asymmetric expansion of the membrane that occurs during the first chlamydial division within infected cells. In addition, FtsQ remains associated with the septum throughout the division process.

A major unresolved question that arose from these studies is how the appearance of bacterial proteins in the daughter cell is regulated. Some cytosolic proteins were only detectable in mature daughter cells, well after the time when an intact membrane appears to separate the mother and daughter cell. While it is possible that the appearance of proteins like Hsp60 and EF-Tu in the daughter cell is dependent upon *de novo* synthesis in the daughter cell, *Chlamydia* encodes a homologue of FtsK [27], which functions as a molecular motor to translocate DNA across membranes in other bacterial systems [30,31]. Whether chlamydial FtsK or other molecular motors translocate DNA and/or proteins across the septum during division remains to be determined. In addition, future studies will investigate when other proteins necessary for chlamydial cell division are recruited to sites of polar growth and the spatial organization of cell division proteins at those sites. Defining the cellular machinery necessary for this novel polarized cell division process may lead to the development of specific therapeutic approaches for eliminating chronic and acute chlamydial infections in humans.

## Materials and Methods

### Antibodies and Reagents

Affinity purified goat polyclonal antibodies directed against the *Chlamydia* major outer membrane protein (MOMP) were purchased from Meridian Life Science. Affinity purified mouse monoclonal antibodies directed against the *E*. *coli* RNA polymerase β subunit, which crossreact with the chlamydial RNA polymerase β subunit, were purchased from Biolegend. Mouse polyclonal antibodies against GFP were purchased from Zymed. Various AlexaFluor conjugated secondary antibodies and Hoechst were obtained from Invitrogen. Glyburide, A22, and carbenicillin were purchased from Sigma Chemicals.

### Cell Culture and *Chlamydia* Infections

HeLa cells (ATCC) grown on coverslips in DMEM containing 10% fetal calf serum were infected with *C*. *trachomatis* serovar L2 (strain 434/Bu). At various times post-infection, cells were rinsed with phosphate buffered-saline (PBS) and fixed by incubation with 3% formaldehyde, 0.045% glutaraldehyde in PBS for 10 minutes and processed for immunostaining. In some instances, glyburide (300nM), AFN1252 (12nM), carbenicillin (1.5μg/ml), penicillin (0.1μg/ml), or A22 (100nM) were added to infected cells for various times prior to fixation. In some instances, HeLa cells infected with *C*. *trachomatis* serovar L2 or *C*. *trachomatis* serovar L2 containing a tetracycline inducible version of GFP-FtsQ were exposed to different culture conditions and the cells were harvested at 48 hours post-infection and the number of inclusion forming units (IFUs) was determined by a limiting dilution assay.

### Localization Analyses

After fixation, cells were rinsed in PBS and permeabilized by incubation in 90% methanol for 10 minutes. Cells were then rinsed with PBS and incubated with primary antibodies and the appropriate AlexaFluor conjugated secondary antibodies prior to analysis by confocal microscopy. Cells were imaged using a Zeiss LSM710 confocal microscope equipped with a 63x or 100x Plan-Apochromat oil immersion lens. The pinhole size was set to 0.5 airy units during image acquisition. In some instances, cells were imaged on a Zeiss AxioImager.M2 microscope equipped with a 100x Plan-Apochromat oil immersion lens. Images were processed with only linear adjustment of brightness and contrast. The filters that were used on the Zeiss LSM 710 and AxioImager.M2 microscopes during image acquisition resulted in no spectral overlap between the various fluorescent channels that were collected. In addition, 4-color spectral beads were added to each of the samples prior to imaging to ensure there was no shift in the x-y plane during the acquisition of different fluorescent channels. Unless otherwise indicated, the images shown in the figures are individual z-slices from a z-stack that extended above and below a cell.

To determine whether pre-entry EBs exhibited an asymmetric distribution of MOMP, Hsp60, and LPS, EBs attached to coverslips were fixed by incubation with 3% formaldehyde, 0.045% glutaraldehyde in PBS for 10 minutes. The cells were then permeabilized with 0.2% saponin prior to staining with MOMP, Hsp60, and LPS antibodies.

### Quantification of Chlamydial Size and Cell Division Intermediates

To quantify chlamydial size at various times post-infection, random fields of infected HeLa cells were imaged on a Zeiss AxiImager.M2 microscope at 2, 4, 8, and 10 hours post-infection. The diameter of all of the MOMP and Hsp60 double-positive cells in the imaged fields was determined using the length tool in the Zeiss Axiovision 4.7 software. The values presented in S1 Table represent the average diameter from 100 cells from two independent experiments.

For the quantification of cell division intermediates, random fields of infected HeLa cells were imaged at 10, 11, 12, and 13 hours post-infection and all of the MOMP and Hsp60 double-positive cells were categorized either as a polarized cell division intermediate (Figs 2A and 4D) or as multiple cells that completed the first division. The multiple cell category included cells in which the mother and daughter cells were the same size and had a similar content and distribution of MOMP, Hsp60, and DNA (Figs 2B and 4D) and inclusions that contained 3 cells. At least 100 inclusions from two independent experiments were analyzed for each time point. A similar approach was used to quantify the effects of penicillin and carbenicillin on the initial polarized cell division of *C*. *trachomatis* and the percentage of *C*. *trachomatis* that contained 2–4 discrete regions of DNA at 10 hours post-infection.

### Immunoblotting Analysis

A lysate was prepared from HeLa cells infected with *C*. *trachomatis* serovar L2 at 24h post-infection and electrophoresed on a NuPAGE 4–12% bis-Tris precast gels and transferred to PVDF blotting membrane (Novex) according to manufacturer instructions (Invitrogen, NY). The lysate was then subjected to immunoblotting analysis with the mouse anti-EF-Tu antiserum. Immunoreactive species were detected using IRDye 800 labeled goat anti-mouse secondary antibodies (Li-Cor Biosciences). The membrane was scanned using Odyssey Infrared Imaging System (Li-Cor; Lincoln, NE) and analyzed by Image Studio-lite Ver 3.1.

### Electron Microscopy

HeLa cells were infected with *C*. *trachomatis* L2 at an MOI of 1 for seventeen hours before fixation in glutaraldehyde. Embedding and processing of 70-80nm sections were performed as described in Ouellette et al. [32]. EM grids were viewed on a JEOL 1200EX transmission electron microscope.

### BODIPY-Ceramide Labeling and Live Imaging of *Chlamydia*

HeLa cells were infected with *C*. *trachomatis* serovar L2 at a multiplicity of infection of 5 and incubated for 10 hours at 37°C. Green fluorescent BODIPY FL C5 ceramide (Invitrogen) was complexed with 0.034% fatty acid free bovine serum albumin (BSA) in 1XHBSS/HEPES as described by the manufacturer. Infected HeLa cells were incubated with 5μM BODIPY-ceramide/ BSA complex at 4°C for 30 min, washed with complete DMEM twice then incubated with complete DMEM/0.7% BSA for one hour to "back-exchange" excess probe from the plasma membrane. In some experiments, cells were infected with *C*. *trachomatis* that contained an anhydrotetracycline (aTc)-inducible plasmid expressing GFP-FtsQ [17]. Cultures were induced with 37pg/ml aTc at 8 hours post-infection then labeled with red fluorescent BODIPY TR C5 ceramide (Invitrogen) complexed to BSA according to manufacturer’s instructions. Cells were imaged using a Zeiss LSM710 confocal microscope equipped with a 63x Plan-Apochromat oil immersion lens, a TempModule, a CO_2_ Module, and a heating device Humidity S1 module; cultures were housed inside the Heating Insert P S1. Alternatively, cells were imaged using a 63x W Plan-Apochromat immersible lens on a Zeiss AxioImager.M2 microscope equipped with heatable Universal Mounting Frame and Objective Heater. Images were collected using an AxioCam MRm camera and deconvolved using Axiovision 4.7 software.

## Supporting Information

S1 Fig Morphology of polarized cell division intermediates. (A) HeLa cells infected with *C*. *trachomatis* serovar L2 were incubated with green BODIPY-ceramide as described in the Materials and Methods and live cells were imaged at 11 hours post-infection. The images shown are 3-dimensional projections of z-stacks that initiated above and extended below the cells during 3 different stages of the polarized cell division process. The stack size of the projections were 3.5μ in total. (B) HeLa cells infected with *C*. *trachomatis* serovar L2 were untreated or incubated with green BODIPY-ceramide or red BODIPY-ceramide as described in the Materials and Methods. Alternatively, HeLa cells infected with *C*. *trachomatis* serovar L2 containing GFP-FtsQ under the control of a tetracycline inducible promoter were untreated or incubated in the presence of 37pg/ml of anhydrotetracycline (+aTc) throughout the infection. Infected cells were harvested at 48 hours post-infection and the effect of the various treatments on the recovery of IFUs was determined. Values in B represent the average of three independent experiments with standard deviations. (C) The polarized cell division intermediates marked by asterisks in Fig 2A (magnified ~2.1x) and Fig 3B (magnified ~2.1x), and the cell marked by an arrow in Fig 4C (magnified ~1.7x) were reoriented to highlight the very similar morphology of dividing cells analyzed by immunofluorescence and electron microscopic techniques. (D) HeLa cells infected with *C*. *trachomatis* were fixed at 13 hours post-infection. The cells were then permeabilized and incubated with rabbit polyclonal antibodies against IncG and goat polyclonal antibodies against MOMP followed by donkey anti-rabbit IgG conjugated to Alexa Fluor 568 and donkey anti-goat IgG conjugated to Alexa Fluor 488. Following washing, the cells were stained with Hoechst 33342 prior to confocal analysis. Arrowhead in D points to the center of the septum that contained lower levels of MOMP. White bar in B is 0.5μ.
(TIF)

S2 Fig Polarized cell division intermediates and polarity in chlamydial RBs and EBs. (A) HeLa cells were infected with *C*. *trachomatis* serovar L2. At 11 hours post-infection, carbenicillin was added to the cells and the cells were subsequently fixed at 16 hours post-infection. The cells were then permeabilized with 90% methanol and incubated with rabbit antibodies against Hsp60 (blue) and goat antibodies against MOMP (green) followed by donkey anti-rabbit IgG conjugated to Alexa Fluor 633 and donkey anti-goat IgG conjugated to Alexa Fluor 488. Following washing, the cells were stained with Hoechst 33342 (red) prior to imaging by epifluorescent microscopy. (B) Purified EBs were fixed and permeabilized with 0.2% saponin then stained with goat antibodies against MOMP, mouse antibodies against LPS, and rabbit antibodies against Hsp60 followed by donkey anti-goat IgG conjugated to Alexa Fluor 488, donkey anti-mouse IgG conjugated to Alexa Fluor 568, and donkey anti-rabbit IgG conjugated to Alexa Fluor 647. The cells were then imaged by confocal microscopy. Hsp60 accumulates in MOMP-poor regions of EBs (marked by arrows). The staining profiles of the EBs shown are representative of images obtained of >100 cells positive for all three markers from two independent experiments. White bars are 0.5μ.
(TIF)

S1 Table Size of *C*. *trachomatis* at various times post-infection. Random fields of infected HeLa cells were imaged at 2, 4, 8, and 10 hours post-infection and the diameter of all of the MOMP and Hsp60 double-positive cells in the imaged fields was determined using the length tool in the Zeiss Axiovision 4.7 software. The values shown are the average from 100 cells from two independent experiments. The diameter of *C*. *trachomatis* at these times post-infection is somewhat larger than the reported values for EBs and RBs obtained from EM studies [33], but they are similar to the diameter of *C*. *trachomatis* reported in previous immunofluorescence analyses [9,11].
(DOCX)

S1 Video 3-D animation illustrating the spatial organization of MOMP, Hsp60, and DNA in *Chlamydia* at an early stage of the polarized cell division process. HeLa cells infected with C. trachomatis were fixed at 11 hours post-infection. Following permeabilization, the cells were incubated with rabbit polyclonal antibodies against Hsp60 (blue) and goat polyclonal antibodies against MOMP (green) followed by donkey anti-rabbit IgG conjugated to Alexa Fluor 568 and donkey anti-goat IgG conjugated to Alexa Fluor 488. The cells were stained Hoechst 33342 (red) prior to analysis by epifluorescent microscopy. The movie is an animation of a 3 dimensional projection of the z-slices that extended above and below the cell. The stack size was 3.5μ in total.
(M4V)

S2 Video 3-D animation illustrating the spatial organization of MOMP, Hsp60, and DNA in *Chlamydia* at an intermediate stage of the polarized cell division process. HeLa cells infected with C. trachomatis were fixed at 11 hours post-infection. Following permeabilization, the cells were incubated with rabbit polyclonal antibodies against Hsp60 (blue) and goat polyclonal antibodies against MOMP (green) followed by donkey anti-rabbit IgG conjugated to Alexa Fluor 568 and donkey anti-goat IgG conjugated to Alexa Fluor 488. The cells were stained Hoechst 33342 (red) prior to analysis by epifluorescent microscopy. The movie is an animation of a 3 dimensional projection of the z-slices that extended above and below the cell. The stack size was 3.5μ in total. UniProtKB gene accession numbers–*C*. *trachomatis* Hsp60, 60 kDa chaperonin GroEL—A0A0H3MAQ6; *C*. *trachomatis* EF-Tu, elongation factor Tu—B0B7N8; *C*. *trachomatis* MOMP, major outer membrane porin—P06597. Accession numbers are for *C*. *trachomatis* serovar L2 (strain 434/Bu).
(M4V)
