# Correction: Polarized Cell Division of *Chlamydia trachomatis*

**DOI:** 10.1371/journal.ppat.1005866

**Published:** 2016-08-30

**Authors:** Yasser Abdelrahman, Scot P. Ouellette, Robert J. Belland, John V. Cox

In the Material and Methods section, the concentrations for glyburide, AFN1252, and A22 are incorrect. The concentrations for glyburide, AFN1252, and A22 should be 300 micromolar, 12 micromolar, and 100 micromolar, respectively.
