# Supplementary figures and images for: Polarized Cell Division of Chlamydia trachomatis
Source: PLoS Pathog. 2016 Aug 9;12(8):e1005822. doi: 10.1371/journal.ppat.1005822 (PMC4978491; doi:10.1371/journal.ppat.1005822)

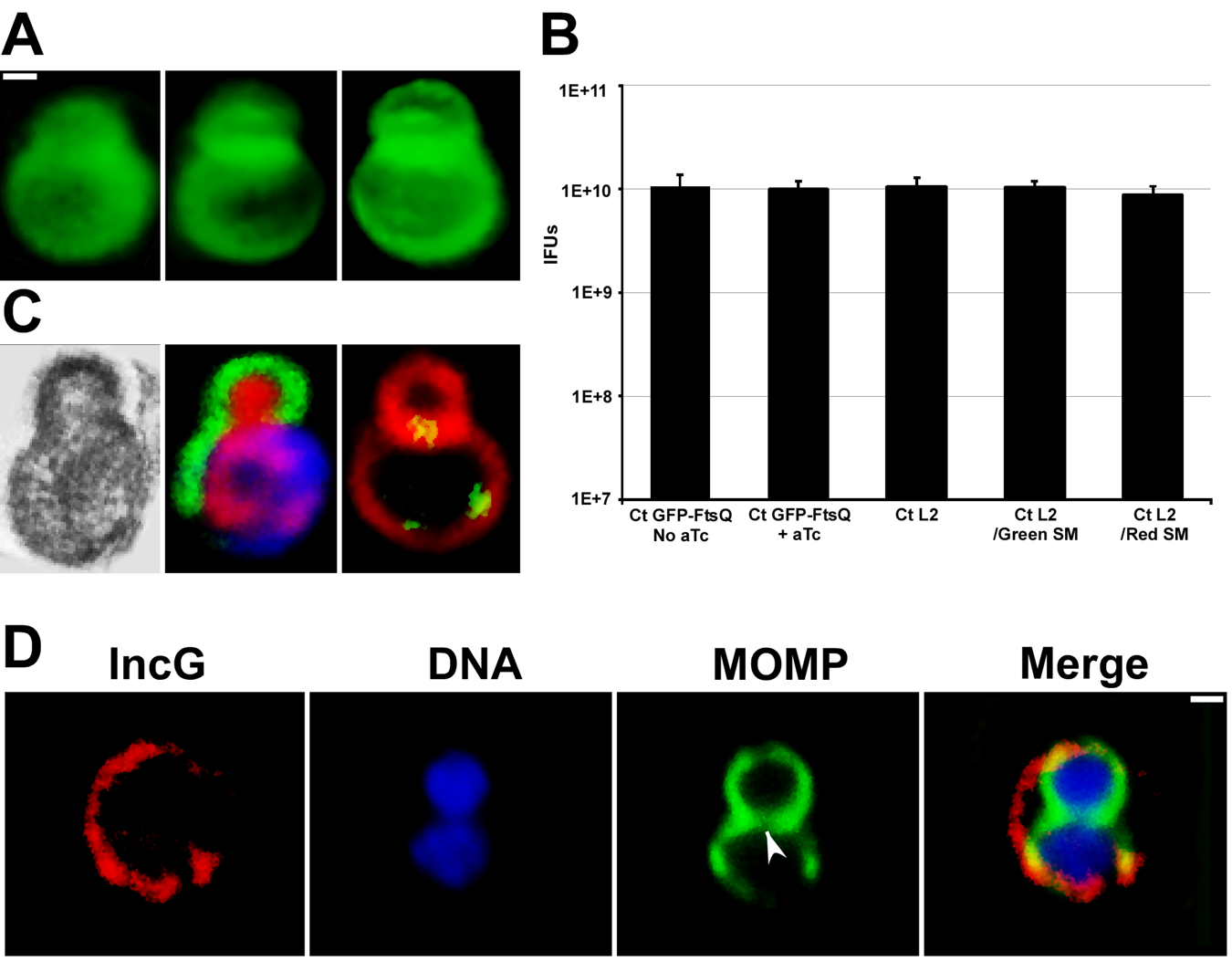

Supplement: S1 Fig — (A) HeLa cells infected with C. trachomatis serovar L2 were incubated with green BODIPY-ceramide as described in the Materials and Methods and live cells were imaged at 11 hours post-infection. The images shown are 3-dimensional projections of z-stacks that initiated above and extended below the cells during 3 different stages of the polarized cell division process. The stack size of the projections were 3.5μ in total. (B) HeLa cells infected with C. trachomatis serovar L2 were untreated or incubated with green BODIPY-ceramide or red BODIPY-ceramide as described in the Materials and Methods. Alternatively, HeLa cells infected with C. trachomatis serovar L2 containing GFP-FtsQ under the control of a tetracycline inducible promoter were untreated or incubated in the presence of 37pg/ml of anhydrotetracycline (+aTc) throughout the infection. Infected cells were harvested at 48 hours post-infection and the effect of the various treatments on the recovery of IFUs was determined. Values in B represent the average of three independent experiments with standard deviations. (C) The polarized cell division intermediates marked by asterisks in Fig 2A (magnified ~2.1x) and Fig 3B (magnified ~2.1x), and the cell marked by an arrow in Fig 4C (magnified ~1.7x) were reoriented to highlight the very similar morphology of dividing cells analyzed by immunofluorescence and electron microscopic techniques. (D) HeLa cells infected with C. trachomatis were fixed at 13 hours post-infection. The cells were then permeabilized and incubated with rabbit polyclonal antibodies against IncG and goat polyclonal antibodies against MOMP followed by donkey anti-rabbit IgG conjugated to Alexa Fluor 568 and donkey anti-goat IgG conjugated to Alexa Fluor 488. Following washing, the cells were stained with Hoechst 33342 prior to confocal analysis. Arrowhead in D points to the center of the septum that contained lower levels of MOMP. White bar in B is 0.5μ. (TIF) [file ppat.1005822.s001.tif]

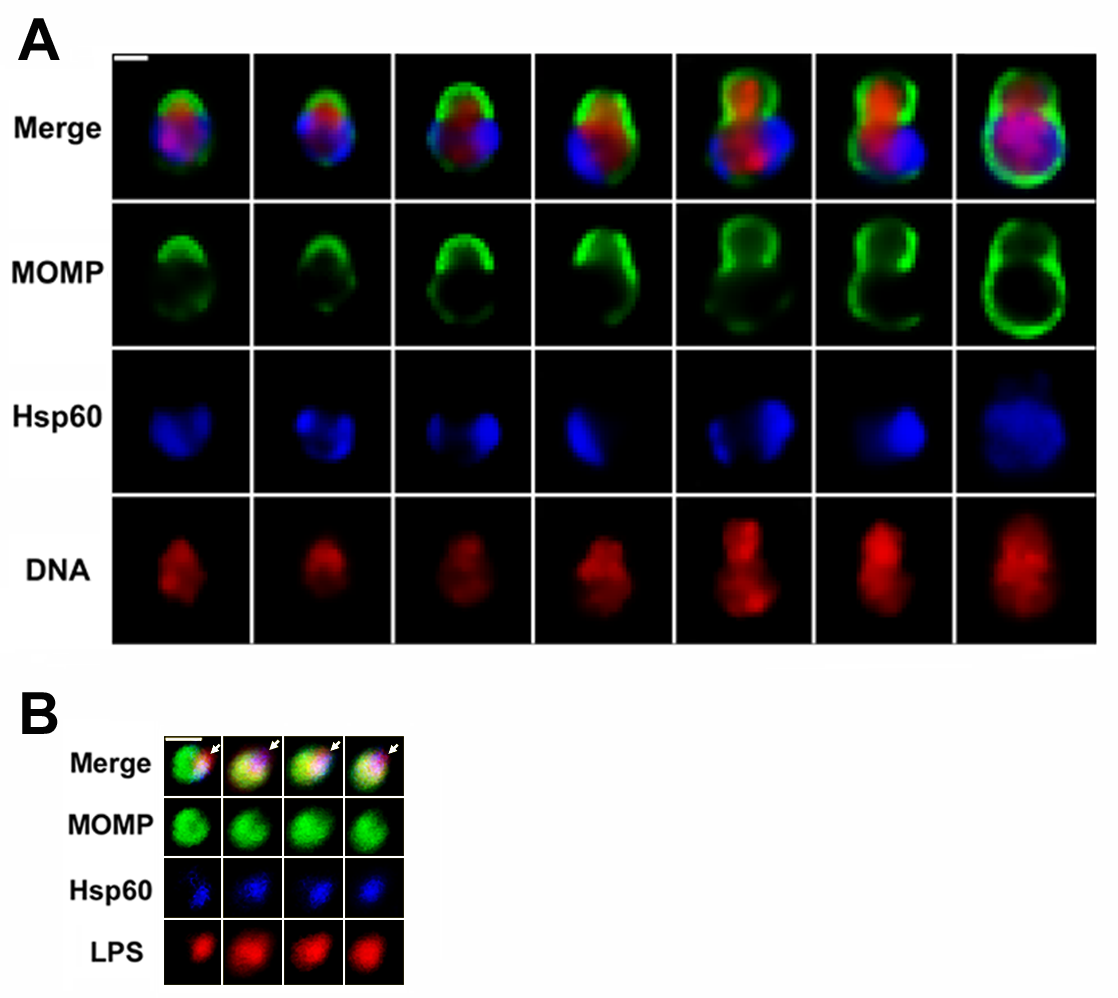

Supplement: S2 Fig — (A) HeLa cells were infected with C. trachomatis serovar L2. At 11 hours post-infection, carbenicillin was added to the cells and the cells were subsequently fixed at 16 hours post-infection. The cells were then permeabilized with 90% methanol and incubated with rabbit antibodies against Hsp60 (blue) and goat antibodies against MOMP (green) followed by donkey anti-rabbit IgG conjugated to Alexa Fluor 633 and donkey anti-goat IgG conjugated to Alexa Fluor 488. Following washing, the cells were stained with Hoechst 33342 (red) prior to imaging by epifluorescent microscopy. (B) Purified EBs were fixed and permeabilized with 0.2% saponin then stained with goat antibodies against MOMP, mouse antibodies against LPS, and rabbit antibodies against Hsp60 followed by donkey anti-goat IgG conjugated to Alexa Fluor 488, donkey anti-mouse IgG conjugated to Alexa Fluor 568, and donkey anti-rabbit IgG conjugated to Alexa Fluor 647. The cells were then imaged by confocal microscopy. Hsp60 accumulates in MOMP-poor regions of EBs (marked by arrows). The staining profiles of the EBs shown are representative of images obtained of >100 cells positive for all three markers from two independent experiments. White bars are 0.5μ. (TIF) [file ppat.1005822.s002.tif]
