# Supplementary material for: Polarized Cell Division of Chlamydia trachomatis
Source: PLoS Pathog. 2016 Aug 9;12(8):e1005822. doi: 10.1371/journal.ppat.1005822 (PMC4978491; doi:10.1371/journal.ppat.1005822)
Supplement: S1 Table — Random fields of infected HeLa cells were imaged at 2, 4, 8, and 10 hours post-infection and the diameter of all of the MOMP and Hsp60 double-positive cells in the imaged fields was determined using the length tool in the Zeiss Axiovision 4.7 software. The values shown are the average from 100 cells from two independent experiments. The diameter of C. trachomatis at these times post-infection is somewhat larger than the reported values for EBs and RBs obtained from EM studies [33], but they are similar to the diameter of C. trachomatis reported in previous immunofluorescence analyses [9,11]. (DOCX) [file ppat.1005822.s003.docx]

| **Time Post Infection** | **Diameter in Microns** |
| --- | --- |
| **2 Hours** | **0.51 ± 0.033** |
| **4 Hours** | **0.73 ± 0.094** |
| **8 Hours** | **1.02 ± 0.14** |
| **10 Hours** | **1.32 ± 0.25** |
